# Recording of hospitalizations for acute exacerbations of COPD in UK electronic health care records

**DOI:** 10.2147/CLEP.S117867

**Published:** 2016-11-21

**Authors:** Kieran J Rothnie, Hana Müllerová, Sara L Thomas, Joht S Chandan, Liam Smeeth, John R Hurst, Kourtney Davis, Jennifer K Quint

**Affiliations:** 1 Respiratory Epidemiology, Occupational Medicine and Public Health, National Heart and Lung Institute, Imperial College London, London, UK; 2 Faculty of Epidemiology and Population Health, London School of Hygiene and Tropical Medicine, London, UK; 3 Respiratory Epidemiology, GlaxoSmithKline R&D, Uxbridge, London; 4 Medical School; 5 UCL Respiratory, University College London, London, UK

**Keywords:** validation, linked data, COPD, hospitalization, cause-specific hospitalization

## Abstract

**Background:**

Accurate identification of hospitalizations for acute exacerbations of chronic obstructive pulmonary disease (AECOPD) within electronic health care records is important for research, public health, and to inform health care utilization and service provision. We aimed to develop a strategy to identify hospitalizations for AECOPD in secondary care data and to investigate the validity of strategies to identify hospitalizations for AECOPD in primary care data.

**Methods:**

We identified patients with chronic obstructive pulmonary disease (COPD) in the Clinical Practice Research Datalink (CPRD) with linked Hospital Episodes Statistics (HES) data. We used discharge summaries for recent hospitalizations for AECOPD to develop a strategy to identify the recording of hospitalizations for AECOPD in HES. We then used the HES strategy as a reference standard to investigate the positive predictive value (PPV) and sensitivity of strategies for identifying AECOPD using general practice CPRD data. We tested two strategies: 1) codes for hospitalization for AECOPD and 2) a code for AECOPD other than hospitalization on the same day as a code for hospitalization due to unspecified reason.

**Results:**

In total, 27,182 patients with COPD were included. Our strategy to identify hospitalizations for AECOPD in HES had a sensitivity of 87.5%. When compared with HES, using a code suggesting hospitalization for AECOPD in CPRD resulted in a PPV of 50.2% (95% confidence interval [CI] 48.5%–51.8%) and a sensitivity of 4.1% (95% CI 3.9%–4.3%). Using a code for AECOPD and a code for hospitalization due to unspecified reason resulted in a PPV of 43.3% (95% CI 42.3%–44.2%) and a sensitivity of 5.4% (95% CI 5.1%–5.7%).

**Conclusion:**

Hospitalization for AECOPD can be identified with high sensitivity in the HES database. The PPV and sensitivity of strategies to identify hospitalizations for AECOPD in primary care data alone are very poor. Primary care data alone should not be used to identify hospitalizations for AECOPD. Instead, researchers should use data that are linked to data from secondary care.

## Background

Chronic obstructive pulmonary disease (COPD) is a common progressive lung disease characterized by airflow obstruction, which is not fully reversible. In the UK, over 1 million people have been diagnosed with COPD, with an estimated further 2 million remaining undiagnosed.^1^,^2^ People with COPD often have periods of acute worsening of symptoms beyond the normal day-to-day variation, which may require a change in the patient’s treatment; these episodes are known as acute exacerbations of COPD (AECOPD). On average, people with COPD experience around two AECOPD every year^3^ (including mild events), and AECOPD are important drivers of morbidity and mortality.^4^–^6^ Most episodes of AECOPD are managed in primary care or by the patient; however, more severe events and/or events in patients with more severe disease or significant comorbidities may require admission to hospital. Hospitalizations for AECOPD are serious events with around 8%^7^ of those admitted dying in hospital and 23% dying within 1 year. As well as being important for individuals, as the second most common reason for emergency admission to hospital in the UK,^8^ they are also of great public health importance. Consequently, hospitalizations for AECOPD are a key outcome in clinical trials and observational studies in people with COPD.

Health care in the UK is mainly provided by the National Health Service (NHS), a public health care system. Primary health care in the NHS is provided by general practitioners (GPs), and over 98% of the UK population are registered with an NHS GP. In the UK, both data from primary care and data related to hospital admissions are readily available and are routinely used for research and for health service planning. With potentially very large sample sizes and representative and detailed real-life data, electronic health care records (EHRs) provide an excellent resource in which to conduct epidemiological studies, including disease epidemiology and comparative safety and effectiveness assessments of interventions. As well as observational studies, an exciting new area in electronic EHRs research is their use for recruitment and follow-up of patients in pragmatic clinical trials,^9^ and these will require valid definitions of important outcomes. In addition to research, EHRs can also be used in areas such as national audits of care and by commissioning groups to plan local services.

However, as routine electronic medical or health care records data are not collected for the purpose of research or audit, one potential limitation of these data is the accuracy and completeness of coded diagnoses. The Clinical Practice Research Datalink (CPRD) is a large database of data from UK primary care. One strength of the CPRD database is its linkages with other databases including Hospital Episodes Statistics (HES) data, an administrative database of all hospital admissions in England.

CPRD has been used extensively for research. Many studies have investigated the validity of CPRD diagnoses for research use, and in general, these have been found to be high.^10^ For specific conditions, the validity of research using CPRD data will depend on both the validity of the algorithm that researchers use to identify the condition and the propensity for the condition to be missed, misrecorded, or misdiagnosed by GPs. Several others have found low completeness when using primary care records alone to identify cause-specific hospitalizations.^11^–^13^ One solution to this problem is to use primary care data, such as CPRD, linked to secondary care data, such as HES. When studying hospitalized AECOPD, for practical reasons, this is not always done,^14^–^16^ and others have called for the recording hospitalizations for AECOPD in UK primary care data to be validated using HES as a reference standard.^17^

Our study had two aims: 1) to investigate sensitivity of recording of hospital admissions for AECOPD in UK secondary care administrative records (HES) and 2) to use linked primary and secondary care data (CPRD–HES) to assess the positive predictive value (PPV) and sensitivity of strategies to identify hospitalizations for AECOPD using primary care data.

## Methods

### Data sources

The CPRD is a very large clinical electronic EHR database of primary EHRs in the UK. It contains information on areas such as diagnoses, prescriptions, and test results, and some lifestyle data, such as smoking status and body mass index. Currently, there are data for over 11 million patients in CPRD, with 4.4 million of these being active patients (representing around 6.9% of the UK population).^18^ Much of the clinical data recorded in CPRD is in the form of Read codes. Read codes are a clinical classification system used to record diagnoses, symptoms, test results, lifestyle factors such as smoking, and other details of consultations. Some information about patient contacts with secondary care, such as referrals, emergency room visits, and hospital admissions may be also captured in CPRD. However, as this requires someone in the GP practice to manually enter such encounters, their recording may be incomplete.

HES is an administrative database containing information on all episodes of admitted patient care in England requiring overnight stay in hospital; these inpatient data used for this study specifically exclude those only seen in accident and emergency department.^19^ Records for hospital admission in HES are split up into “finished consultant episodes (FCE)”, each of these represent an episode of care under a single consultant. Each hospital admission may be made up of several FCEs. FCE records contain information on up to 20 diagnoses recorded during that episode and are recorded using International Classification of Diseases (ICD, tenth edition) codes. As well as recording the reason for hospitalization, diagnoses recorded in HES may relate to coexistent comorbidities. In addition, there is a financial incentive for hospitals to accurately record comorbidities during each hospitalization. The diagnostic code in the first position in the first FCE is commonly taken to be the reason for hospitalization.

CPRD practices based in England are eligible to be linked with HES data. Approximately 75% of English practices have consented to participate in this linkage, which equates to around 60% of the total CPRD population.^18^ Although much of CPRD is eligible to be linked, HES data are not automatically made available to researchers as obtaining linked data requires further approvals. Many researches may therefore only use “standalone” primary care CPRD data.

### Study population

The total study population consisted of patients in CPRD who had a validated diagnosis of COPD and who were eligible for linkage to HES. Briefly, COPD patients were aged 35 years or older, current or ex-smokers, had a validated diagnostic code suggesting COPD, and at least two prescriptions for a COPD medicine, one within 4 weeks of COPD diagnosis.^20^ Patients were followed up from January 1, 2004, their date of COPD diagnosis, 35th birthday, or CPRD practice “up to standard” date, whichever was latest, to March 31, 2014, date of death, transfer out of practice, or practice last collection date, whichever was earliest.

#### Recording of hospitalizations for AECOPD in HES

**Figure 1:**
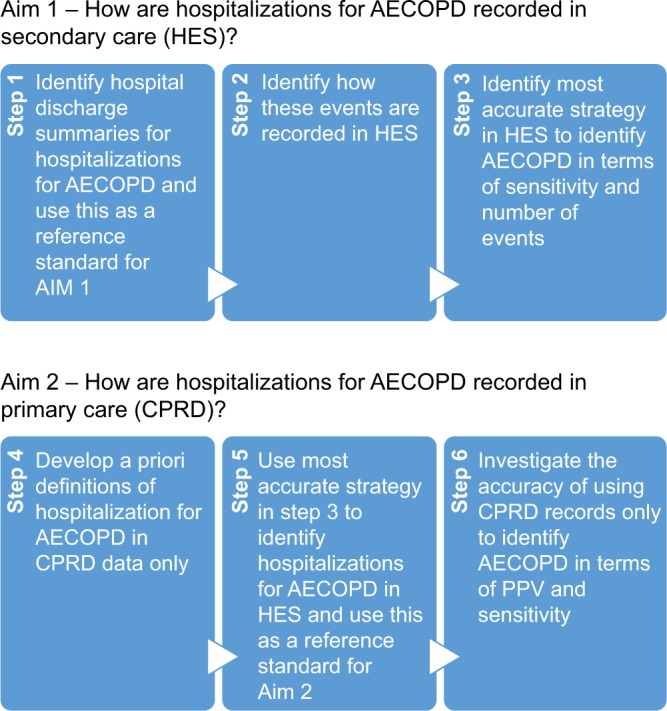
Summary of the methods for each of the aims of the study. **Abbreviations:** AECOPD, acute exacerbations of chronic obstructive pulmonary disease; CPRD, Clinical Practice Research Datalink; HES, Hospital Episodes Statistics; PPV, positive predictive value.

A summary of the analytical approaches for each of the aims is presented in Figure 1. For the first aim, we used hospital discharge summaries to identify how hospitalizations for AECOPD are recorded in HES. Hospital discharge summaries were available for a subset of patients (N=40) who were also included in two previous validation studies (one validating the recording of COPD and one validating the recording of AECOPD in CPRD).^20^,^21^ As part of these studies, GPs were contacted and asked to send material related to their patient’s COPD, including hospital discharge summaries, to investigators. Additional questionnaire data were available for 637 patients, of whom 40 had linkable HES data and discharge letters for an admission to hospital for AECOPD. We therefore obtained 40 discharge summaries for 40 patients who had linkable HES data. We used these summaries as a reference standard to estimate the sensitivity of the possible HES strategies to identify hospitalizations for AECOPD. First, ICD codes which could be used to record hospitalizations for AECOPD in HES were prespecified: “J44.0” and “J44.1” as specific AECOPD codes, the code for lower respiratory tract infection (LRTI) “J22” and the code for COPD “J44.9”. Next, we visualized the diagnostic position of each of the ICD codes used that might potentially be used to record hospitalizations for AECOPD. Then, we used these codes to create strategies that might relate to hospitalizations for AECOPD based on combinations of these codes being in the first position or in any position of FCE (Table 1). We then estimated the sensitivity of each of these strategies in identifying hospitalizations for AECOPD using hospital discharge summaries as the reference standard. Finally, we calculated the total number of events each of these strategies would identify if they were used in the sample.

#### Recording of hospitalizations for AECOPD in CPRD

For the second aim, we identified strategies that might be used to identify hospitalizations for AECOPD in standalone primary care records. Broadly, there were two strategies: 1) presence of a code that suggested hospitalization for AECOPD and 2) presence of a code or codes on the same day, which suggested that the patient both had an AECOPD and had been admitted to hospital. To identify records for AECOPD in CPRD, we used our previously validated algorithm.^21^ We did not include codes suggesting pneumonia in either of these strategies, as although AECOPD may be (incorrectly) coded using these codes, they are unlikely to be used in a strategy to identify hospitalizations for AECOPD for research purposes. Furthermore, we searched the Read code dictionary for codes that suggested hospitalization for AECOPD or for hospitalization without a specified reason. These strategies are summarized in Table 2. We also removed dates that were coded as COPD “annual review” dates as we have previously demonstrated that AECOPD codes are used at these times despite these not being acute episodes of AECOPD.^21^

To test the validity of different strategies to identify hospitalizations for AECOPD in primary care data, we calculated the PPV and sensitivity of strategies listed in Table 2 using HES-recorded hospitalization for AECOPD as the reference standard. For the estimation of PPV, we looked backward in the HES record for 30 days following a potential AECOPD hospitalization in CPRD; for sensitivity, we looked forward in the CPRD patient record 30 days after the HES-recorded hospital admission to allow for any delays in recording in the GP surgery. As an additional analysis, we increased this window to 60 days. We repeated these analyses stratified by different predefined definitions of HES-recorded hospitalization for AECOPD (definitions 1, 3, and 5 in Table 1).

We conducted an additional analysis to investigate other ways in which hospitalizations for AECOPD may be coded that would not have been picked up by either of the strategies that we developed. To accomplish this goal, we investigated the PPV and sensitivity of just using either a code or codes which suggested the patient had an AECOPD or had been to hospital in identifying hospitalizations for AECOPD (eg, “admission to hospital” alone or “LRTI” alone), ie, when there was information that the COPD patient had either a) been to hospital for an unspecified reason or b) had an AECOPD but no code to suggest that the patient had been to hospital. As admission to hospital may also be recorded by GPs using “consultation types” and “referral types” rather than separate Read codes, we also extended the CPRD definition of an AECOPD code on the same day as a hospitalization code to include these consultation types and referral types, and then assessed this extended definition against our main HES definition of hospitalization for AECOPD. In addition, we also explored a random sample of 100 Read codes present on days in which there was a record for a hospitalization for AECOPD in HES and were not associated with codes for AECOPD or hospitalizations. Statistical analysis was conducted in Stata 14.1 MP (StataCorp LP, College Station, TX, USA) and R 3.2.3.

#### Ethics approval

Ethics approval was obtained from the London School of Hygiene and Tropical Medicine Observational Research Ethics Committee (approval number 6481) and the CPRD Independent Scientific Advisory Committee (approval number 13_116A). Patient records and questionnaire responses were deidentified and anonymized by CPRD staff before being sent to the investigators. The Independent Scientific Advisory Committee protocol is available on request. Further patient consent was not required due to the nature of the study.

## Results

**Figure 2:**
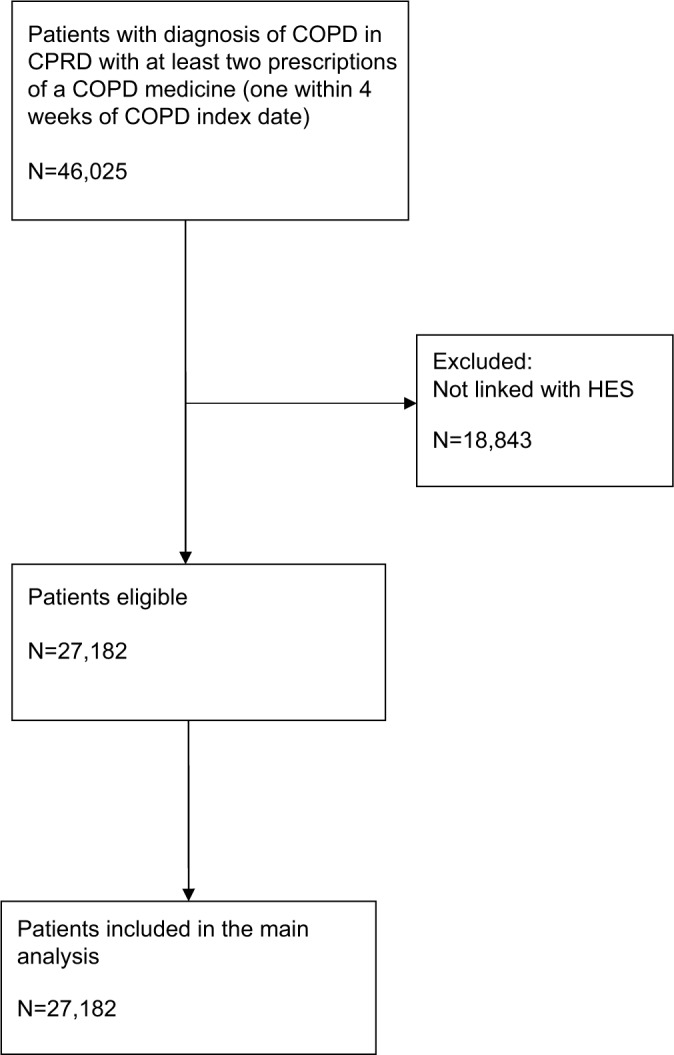
Flow of patients through the study. **Abbreviations:** COPD, chronic obstructive pulmonary disease; CPRD, Clinical Practice Research Datalink; HES, Hospital Episodes Statistics.

In total, 27,182 COPD patients with linked HES–CPRD data were included in the initial cohort after fulfilling inclusion criteria (Figure 2). The characteristics of patients included in the study are summarized in Table 3. In the total cohort, the mean age was 65.5 years (standard deviation: 11.1), 46.5% were females, and 59.7% current smokers. About 54.4% had moderate-to-severe dyspnea (Medical Research Council ≥3), and 36.4% had GOLD grade of airflow limitation 3 or higher.

### Recording of hospitalizations for AECOPD in HES

**Figure 3:**
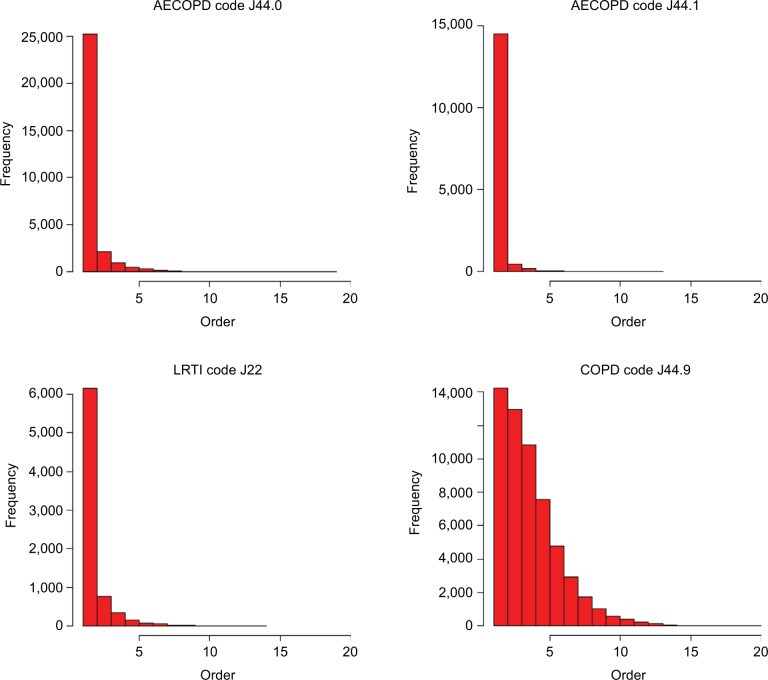
Diagnostic positions of ICD codes for AECOPD, LRTIs, and COPD in Hospital Episodes Statistics records for hospitalizations for COPD patients. **Abbreviations:** ICD, International Classification of Diseases; COPD, chronic obstructive pulmonary disease; AECOPD, acute exacerbations of COPD; LRTI, lower respiratory tract infection.

Graphs demonstrating the diagnostic positions of ICD codes in HES for AECOPD, LRTI, and COPD in FCE for hospitalized COPD patients are shown in Figure 3. These graphs demonstrate that codes for AECOPD and LRTI tend to be used in the first position. The code for COPD, although it is commonly used in the first position, is also often used in subsequent positions.

The findings for the investigation of the validity of the strategies used to identify hospitalizations for AECOPD are presented in Table 4. For the assessment of sensitivity, 40 discharge letters were available. The lowest estimated sensitivity was definition 6, using only a specific AECOPD code in the first position in the first FCE for a hospitalization (sensitivity 65.0%, 95% confidence interval [CI] 45.8%–78.6%). The highest estimated sensitivity was definition 5, using either a specific AECOPD code or an LRTI code in any position or a COPD code in the first position in any FCE during a hospitalization (sensitivity: 87.5%, 95% CI: 72.4%–94.9%).

### Recording of hospitalizations for AECOPD in primary care records

Using the most sensitive definition of AECOPD hospitalization identified in HES as the reference standard, the PPV for the specific AECOPD hospitalization code in CPRD was 50.2% (95% CI: 48.5%–51.8%) and the sensitivity was 4.1% (95% CI: 3.9%–4.3%) (Table 5). Using AECOPD identified using the previously validated algorithm on the same day as a Read code suggesting hospitalization due to unspecified reason in the primary care record resulted in a PPV of 43.3% (95% CI: 42.3%–44.2%) and a sensitivity of 5.4% (95% CI: 5.1%–5.7%). The use of different HES definitions of hospitalization for AECOPD did not result in markedly different results (Table 5). The results of the additional analysis repeated using only the day of the HES recorded event and using a 60-day window rather than a 30-day window following the HES recorded event are presented in the (Tables S1–S5). With the exception of an increase in the sensitivity of use of AECOPD code alone or nonspecific hospitalization code alone as the window was increased, these results did not differ significantly from the analysis using a 30-day window.

When the definition using AECOPD codes on the same day as hospitalization codes was extended to use consultation or referral types indicating hospitalization, this reduced the PPV to 14.6% (95% CI: 14.2%–14.9%) and increased the sensitivity to 6.0% (95% CI: 5.7%–6.3%). In the additional analysis to investigate the use of either a code or codes suggesting AECOPD or hospitalization for any reason, the use of the AECOPD algorithm alone resulted in a PPV of 1.8% (95% CI: 1.7%–1.8%) and a sensitivity of 34.2% (95% CI: 33.7%–34.6%). The use of a code suggesting hospitalization alone resulted in a PPV of 14.5% (95% CI: 14.3%–14.6%) and a sensitivity of 53.5% (95% CI: 53.0%–54.0%). These results repeated using different HES definitions for hospitalization due to AECOPD are presented in the (Tables S1, S3 and S5).

When assessing a random sample of 100 Read codes on the day of admission on which patients had a HES hospitalization for AECOPD (after excluding codes that either suggested AECOPD according to our algorithm, or hospitalization for any reason), we found many of these related to nonspecific Read Terms suggesting patient contact such as “had a chat to patient”, “patient reviewed”, and “seen in out-of-hours center” (N=41); several related to recording of either heart rate or blood pressure (N=16); some related to contact with secondary care (but not necessarily suggesting admission to hospital), such as “seen by respiratory physician” or “letter from specialist” (N=10); few related to symptoms of an AECOPD such as “cough” (N=5); the remaining (N=28) were not specific for AECOPD.

## Discussion

We developed a valid strategy to identify hospitalizations for AECOPD using HES-linked CPRD data. Using this definition as a reference standard, we found that using information from primary care data alone resulted in low PPV and sensitivity for identifying hospitalizations for AECOPD.

When we assessed the validity of the recording of hospitalizations for AECOPD in HES, we found that the most sensitive strategy was the use of a specific AECOPD or LRTI ICD-10 code in any position in any FCE, or the COPD ICD-10 code in the first position only in any FCE in a hospitalization (sensitivity 87.5%). The use of the COPD ICD-10 code in any position results in a very large number of events, and this likely represents it being used to record COPD as a comorbidity not as a reason for hospitalization. Although the exact definition used in future studies may differ depending on the needs of the study, this definition is likely to represent the “optimal” way to identify hospitalizations for AECOPD in HES. Restricting the definition to the specific AECOPD codes in the first position only in the first FCE reduced the sensitivity to around 65%. The failure to recognize the remaining patients is likely to represent COPD patients receiving a nonspecific ICD-10 code such as “shortness of breath” on an assessment ward before being moved to a specialist ward.

For the analysis of the accuracy of using primary care data only to identify hospitalized AECOPD, using the most sensitive HES definition of AECOPD as the reference standard, the maximum PPV achievable was 50.2% and the maximum sensitivity achievable was only 5.4%. The use of such strategies to identify hospitalizations for AECOPD would mean that the vast majority of “true” events would not be picked up, and that of those events which were picked up, only half would be “true” events. The findings from our additional analysis suggest that GPs are recording the majority of AECOPD hospitalizations simply by using generic hospitalization codes and/or AECOPD codes alone. The use of consultation and referral type data increased the sensitivity very slightly, but resulted in a large decrease in PPV. Although the use of nonspecific hospitalization codes or AECOPD codes alone had a higher sensitivity, particularly when the window was extended to 60 days, the PPV was very low and it is unclear if these relate to the index HES recorded event or further moderate AECOPD or hospitalizations. For the other CPRD definitions of AECOPD hospitalization, increasing the window beyond 60 days may have improved performance, but it would become difficult to differentiate multiple hospitalizations from each other. The findings from the examination of Read codes on days on which AECOPD hospitalizations occurred but were not identified by any of the CPRD strategies suggest that on the day of hospitalization, many AECOPD hospitalizations are also recorded using even less specific codes than a generic hospitalization code. This is of clinical concern given the impact of first, and subsequent, admission to hospital for AECOPD on prognosis in COPD patients.^22^

Our finding that validity of primary care recorded hospitalizations for AECOPD is low is certainly striking, but perhaps not surprising. Previous works in cause-specific hospitalization in other disease areas have produced similar results. Recent studies investigating the validity and completeness of UK primary care recording of admission to hospital for acute myocardial infarction,^13^ poisonings, fractures and burns,^11^ and gastrointestinal bleeding^12^ have all found that strategies to identify these events in primary care tend to have low–moderate sensitivity and varying levels of PPV. In addition, a recent study showed that using HES-linked CPRD data, rather than CPRD data alone, resulted in a doubling of incidence of community-acquired pneumonia and that this could be attributable to patients presenting directly to hospital without first consulting their GP.^23^ These findings are consistent with our results. A recent study did find a high PPV for codes suggesting hospital admission for community-acquired pneumonia in the general population, but this was only after restricting for those with a recent nonspecific respiratory infection code, and this study did not assess sensitivity.^24^ Interestingly, another study in UK primary care records found an increasing trend toward coding episodes of influenza-like illness (ILI) using nonspecific codes rather than definite ILI codes, and a tendency not to use definite ILI codes in populations in whom there was more likely to be diagnostic uncertainty.^25^ These findings are reflected in our results. The reasons that the PPV and sensitivity of the recording in primary care of hospitalizations for AECOPD are particularly low are likely to be: the use of nonspecific codes, diagnostic uncertainty, and the use of apparently acute codes to record historical events. Our findings from this analysis are in stark contrast to our validation of the recording of AECOPD treated in general practice, where we found high PPV and adequate sensitivity.^21^

EHRs are becoming increasingly used both for research and for audit and service planning. Due to its universal public health care system, the UK is an attractive setting to use electronic EHRs to study diseases and medical interventions. Although GPs should be informed when their patients are admitted to hospital, this may not be recorded in such a way that is useful for researchers. Just as details such as comorbidities, prior medicine use, and sociodemographic details might be missing from secondary care records, detailed information about hospital admissions may be missing from primary care records. The present study underlines previous findings that hospital admission diagnoses and procedures are not consistently recorded in primary care. Although this may reduce sample sizes and result in a lag in available linked data, it seems that, for some conditions, use of primary care data alone may not result in valid definitions when used to study events, which may result in admission to hospital. Although the validity of definitions will likely differ between different conditions, researchers should be cautious about using primary care data alone to define cause-specific hospitalizations.

The major strength of this study is the size and representativeness of the sample. We used data for over 27,182 COPD patients. Our assessment of the validity of the HES definitions of AECOPD hospitalization was only based on 40 patients, however, which may have affected the precision of the sensitivity estimates for the HES definitions. We also made use of a validated strategy to identify patients with COPD in the CPRD. Although there is some uncertainty in the best definition of hospitalization for AECOPD in HES to use as the reference standard, we used hospital discharge summaries to validate how these were recorded in HES. In addition, we repeated our main analysis using several different HES definitions of hospitalization for AECOPD, and these did not change our conclusions.

One weakness of the study is that the HES strategy did not identify all the hospitalizations for AECOPD and that we could not assess the PPV of the HES strategy; however, in the main analysis, we used a strategy with a sensitivity of 87.5%, and this is unlikely to have impacted on the conclusions of the study. In addition, although we were able to assess the sensitivity of the strategies to identify hospitalizations for AECOPD in HES, we were unable to assess their PPV. Imperfect PPV of the definition of hospitalizations for AECOPD in HES would have the effect of underestimating the sensitivity of CPRD algorithms. Using a range of hypothetical PPVs, we can estimate the potential effect of lower PPV of the HES definitions by multiplying the estimated sensitivity of the CPRD definitions by the inverse of the PPV (1/hypothetical PPV). For example, if the PPV of our main HES definition was only 80%, the sensitivity of the CPRD definition using AECOPD hospitalization codes would only rise from 4.1% to 5.1%; and the algorithm using an AECOPD code and a hospitalization code on the same day would rise to 6.8%. Even in the unlikely situation that the PPV of our main HES algorithm was as low as 60%, the respective sensitivities would only increase to 6.8% for an AECOPD hospitalization code and 9.0% for an AECOPD code and hospitalization code on the same day. We also assessed the CPRD definitions of hospitalization for AECOPD using several definitions of AECOPD hospitalizations in HES, and the findings did not change when we used definitions with varying sensitivities. The impact of this limitation is therefore likely to be small.

## Conclusion

In the UK, primary care EHR data should not be used alone to identify hospitalizations for exacerbations of COPD. To accurately identify hospitalizations for AECOPD and to correctly classify AECOPD either as those treated in primary care or resulting in hospitalization, researchers should use primary care data linked with secondary care data on hospitalizations.

## Supplementary material

**Table S1:** PPV and sensitivity of CPRD strategies to identify hospitalizations for AECOPD using different HES definitions as reference standard using day of admission in HES only

HES AECOPD definition	CPRD strategy	PPV (95% CI)	Sensitivity (95% CI)
AECOPD hospitalization or LRTI code in any position or COPD code in the first position in any FCE	AECOPD identified using algorithm	0.7% (0.7–0.7)	7.2% (6.95–7.6)
	Nonspecific hospitalization code	10.3% (10.1–10.6)	27.1% (26.5–27.7)
Either specific AECOPD code in any position or COPD code in the first position	AECOPD identified using algorithm	0.6% (0.6–0.6)	7.2% (6.9–7.6)
	Nonspecific hospitalization code	9.1% (8.9–9.3)	27.6% (27.0–28.2)
Either specific AECOPD code in the first position in any FCE	AECOPD identified using algorithm	0.6% (0.6–0.6)	7.4% (7.1–7.9)
	Nonspecific hospitalization code	8.7% (8.5–8.9)	28.1% (27.4–28.7)

**Abbreviations:** CI, confidence interval; COPD, chronic obstructive pulmonary disease; AECOPD, acute exacerbations of COPD; LRTI, lower respiratory tract infection; HES, Hospital Episodes Statistics; FCE, finished consultant episodes; CPRD, Clinical Practice Research Datalink; PPV, positive predictive value.

**Table S2:** PPV and sensitivity of CPRD strategies to identify hospitalizations for AECOPD using different HES definitions as reference standard using day of admission in HES only

HES AECOPD definition	CPRD strategy	PPV (95% CI)	Sensitivity (95% CI)
AECOPD hospitalization code or LRTI code in any position or COPD in the first position in any FCE	AECOPD hospitalization code	47.6% (44.3–50.8)	1.9% (1.7–2.0)
	AECOPD identified using validated algorithm and hospitalization code	41.9% (39.8–44.0)	3.7% (3.5–4.0)
Either specific AECOPD code in any position or COPD code in the first position	AECOPD hospitalization code	43.6% (40.5–46.9)	2.1% (1.9–2.3)
	AECOPD identified using validated algorithm and hospitalization code	36.8% (34.7–38.9)	3.7% (3.5–4.0)
Either specific AECOPD code in the first position in any FCE	AECOPD hospitalization code	46.5% (44.9–48.2)	2.1% (1.9–2.3)
	AECOPD identified using validated algorithm and hospitalization code	35.3% (33.2–37.4)	3.8% (3.5–4.1)

**Abbreviations:** CI, confidence interval; COPD, chronic obstructive pulmonary disease; AECOPD, acute exacerbations of COPD; LRTI, lower respiratory tract infection; HES, Hospital Episodes Statistics; FCE, finished consultant episodes; CPRD, Clinical Practice Research Datalink; PPV, positive predictive value.

**Table S3:** PPV and sensitivity of record of AECOPD or nonspecific hospitalization code to identify hospitalizations for AECOPD using different HES definitions as reference standard allowing 30 days after HES record of hospitalization for AECOPD

HES AECOPD definition	CPRD strategy	PPV (95% CI)	Sensitivity (95% CI)
AECOPD hospitalization code or LRTI code in any position or COPD code in first position in any FCE	AECOPD identified using algorithm	1.8% (1.7–1.8)	34.2% (33.7–34.6)
	Nonspecific hospitalization code	14.5% (14.3–14.6)	53.5% (53.0–54.0)
Either specific AECOPD code in any position or COPD code in the first position	AECOPD identified using algorithm	1.5% (1.5–1.6)	34.6% (34.1–35.1)
	Nonspecific hospitalization code	12.6% (12.5–12.8)	54.1% (53.6–54.6)
Either specific AECOPD code in the first position in any FCE	AECOPD identified using algorithm	1.5% (1.4–1.5)	35.1% (34.6–35.6)
	Nonspecific hospitalization code	12.0% (11.9–12.2)	54.8% (54.3–55.3)

**Abbreviations:** CI, confidence interval; COPD, chronic obstructive pulmonary disease; AECOPD, acute exacerbations of COPD; LRTI, lower respiratory tract infection; HES, Hospital Episodes Statistics; FCE, finished consultant episodes; CPRD, Clinical Practice Research Datalink; PPV, positive predictive value.

**Table S4:** PPV and sensitivity of CPRD strategies to identify hospitalizations for AECOPD using different HES definitions as reference standard allowing 60 days after HES record of hospitalization for AECOPD

HES AECOPD definition	CPRD strategy	PPV (95% CI)	Sensitivity (95% CI)
AECOPD hospitalization code or LRTI code in any position or COPD in the first position in any FCE	AECOPD hospitalization code	50.7% (49.1–52.3)	4.4% (4.3–4.6)
	AECOPD identified using validated algorithm and hospitalization code	46.1% (44.3–47.8)	6.1% (5.8–6.4)
Either specific AECOPD code in any position or COPD code in the first position	AECOPD hospitalization code	49.5% (48.0–51.1)	5.0% (4.8–5.3)
	AECOPD identified using validated algorithm and hospitalization code	41.2% (39.5–42.9)	6.2% (5.9–6.6)
Either specific AECOPD code in the first position in any FCE	AECOPD hospitalization code	46.6% (45.0–48.2)	5.0% (4.8–5.3)
	AECOPD identified using validated algorithm and hospitalization code	39.5% (37.8–41.2)	6.4% (6.1–6.8)

**Abbreviations:** CI, confidence interval; COPD, chronic obstructive pulmonary disease; AECOPD, acute exacerbations of COPD; LRTI, lower respiratory tract infection; HES, Hospital Episodes Statistics; FCE, finished consultant episodes; CPRD, Clinical Practice Research Datalink; PPV, positive predictive value.

**Table S5:** PPV and sensitivity of record of AECOPD or nonspecific hospitalization code to identify hospitalizations for AECOPD using different HES definitions as reference standard allowing 60 days after HES record of hospitalization for AECOPD

HES AECOPD definition	CPRD strategy	PPV (95% CI)	Sensitivity (95% CI)
AECOPD hospitalization code or LRTI code in any position or COPD in the first position in any FCE	AECOPD identified using algorithm	2.3% (2.3–2.3)	46.3% (45.8–46.7)
	Nonspecific hospitalization code	14.5% (14.4–14.7)	55.9% (55.4–56.4)
Either specific AECOPD code in any position or COPD code in the first position	AECOPD identified using algorithm	2.0% (2.0–2.1)	46.8% (46.3–47.3)
	Nonspecific hospitalization code	12.7% (12.5–12.9)	56.5% (56.0–57.0)
Either specific AECOPD code in the first position in any FCE	AECOPD identified using algorithm	1.9% (1.9–1.9)	47.3% (46.8–47.8)
	Nonspecific hospitalization code	12.1% (11.9–12.2)	57.2% (56.6–57.7)

**Abbreviations:** CI, confidence interval; COPD, chronic obstructive pulmonary disease; AECOPD, acute exacerbations of COPD; LRTI, lower respiratory tract infection; HES, Hospital Episodes Statistics; FCE, finished consultant episodes; CPRD, Clinical Practice Research Datalink; PPV, positive predictive value.

## Figures and Tables

**Table 1:** Strategies for identifying admissions to hospital for AECOPD in HES

HES definition of AECOPD hospitalization
1. Specific AECOPD code or COPD code in any position in any FCE during spell
2. Specific AECOPD code in any position or COPD code in first position in any FCE during spell
3. Specific AECOPD code in any position in any FCE during spell
4. Specific AECOPD code in any position in or LRTI code or COPD code in first position in any FCE during spell
5. Specific AECOPD code or LRTI code in any position or COPD code in first position in any FCE during spell
6. Specific AECOPD code in first position in first FCE during spell

**Abbreviations:** COPD, chronic obstructive pulmonary disease; AECOPD, acute exacerbations of COPD; LRTI, lower respiratory tract infection; HES, Hospital Episodes Statistics; FCE, finished consultant episodes.

**Table 2:** Possible strategies for identifying hospitalizations for AECOPD using primary care data alone

Definition	Example
Diagnostic code or codes suggesting hospitalization for AECOPD	“Admit COPD emergency”
Diagnostic code(s) suggesting AECOPD (using our previously validated algorithm) and nonspecific code(s) suggesting admission to hospital on the same day	“Acute LRTI” and “admission to hospital” on the same day

**Abbreviations:** COPD, chronic obstructive pulmonary disease; AECOPD, acute exacerbations of COPD; LRTI, lower respiratory tract infection.

**Table 3:** Characteristics of patients included in the study

Characteristic	Overall	Those with hospital discharge information

	N=27,182 (N [%])	N=40 (N [%])
Age group (years)		
≤55	5,003 (18.4)	7 (17.5)
55–64	7,746 (28.5)	16 (40.0)
65–74	8,537 (31.4)	12 (30.0)
≥75	5,896 (21.7)	5 (12.5)
Sex		
Male	14,556 (53.6)	18 (45.0)
MRC breathlessness scale (kg/m^2^; N=21,151)		
<3	9,645 (45.6)	21 (53.8)
≥3	11,506 (54.4)	18 (46.2)
BMI (N=26,447)		
<19	1,441 (5.5)	1 (2.5)
19–25	9,568 (36.2)	18 (45.0)
≥25	15,438 (58.4)	21 (52.5)
GOLD 2006 grade (N=14,055)		
1	2,829 (20.1)	4 (16.7)
2	6,116 (43.5)	6 (25.0)
3	4,075 (29.0)	10 (41.7)
4	1,035 (7.4)	4 (16.7)
Smoking status		
Ex-smoker	10,963 (40.3)	19 (47.5)
Current smoker	16,219 (59.7)	21 (52.5)
Index of multiple deprivation quintile (N=25,852)		
1 (least deprived)	3,632 (14.1)	8 (20.0)
2	5,259 (20.3)	7 (17.5)
3	4,989 (19.3)	7 (17.5)
4	5,794 (22.4)	6 (15.0)
5 (most deprived)	6,178 (23.9)	12 (30.0)

**Abbreviations:** MRC, Medical Research Council; BMI, body mass index; GOLD, Global Initiative for Chronic Obstructive Lung Disease.

**Table 4:** Validity of HES definitions of AECOPD hospitalization

	Discharge summary analysis		Full HES sample analysis

HES definition of AECOPD hospitalization	Number of discharge summary-confirmed AECOPD hospitalizations identified using strategy (N=40 events in 40 patients from discharge letters)	Sensitivity (95% CI) (% of discharge summary-confirmed AECOPD hospitalizations picked up) (N=40 events in 40 patients from discharge letters)	Number of potential AECOPD hospitalization events in total sample identified using strategy (full HES sample for all 27,182 COPD patients included in the study)^a^
Specific AECOPD code or LRTI code in any position or COPD code in the first position in any FCE during spell	35/40	87.5% (72.4%–94.9%)	40,174
Specific AECOPD code or COPD code in any position in any FCE during spell	34/40	85.0% (69.6%–93.3%)	74,590
Specific AECOPD code in any position or LRTI code or COPD code in the first position in any FCE during spell	34/40	85.0% (69.6%–93.3%)	37,966
Specific AECOPD code in any position or COPD code in the first position in any FCE during spell	31/40	77.5% (61.3%–88.2%)	35,793
Specific AECOPD code in any position in any FCE during spell	31/40	77.5% (61.3%–88.2%)	33,933
Specific AECOPD code in the first position in first FCE during spell	26/40	65.0% (48.5%–78.6%)	21,387

**Note:**

a These potential events will represent both true and false positives.

**Abbreviations:** CI, confidence interval; COPD, chronic obstructive pulmonary disease; AECOPD, acute exacerbations of COPD; LRTI, lower respiratory tract infection; HES, Hospital Episodes Statistics; FCE, finished consultant episodes.

**Table 5:** PPV and sensitivity of CPRD strategies to identify hospitalizations for AECOPD using different HES definitions as reference standard allowing 30 days after HES record of hospitalization for AECOPD

HES AECOPD definition	CPRD strategy	PPV (95% CI)	Sensitivity (95% CI)
AECOPD hospitalization or LRTI code in any position or COPD in the first position in any FCE	AECOPD hospitalization code	50.2% (48.5%–51.8%)	4.1% (3.9%–4.3%)
	AECOPD identified using validated algorithm and hospitalization code	43.3% (42.3%–44.2%)	5.4% (5.1%–5.7%)
Either specific AECOPD code in any position or COPD code in the first position	AECOPD hospitalization code	49.0% (47.3%–50.6%)	4.6% (4.5%–4.9%)
	AECOPD identified using validated algorithm and hospitalization code	38.5% (37.6%–39.4%)	5.5% (5.2%–5.9%)
Either specific AECOPD code in the first position in any FCE	AECOPD hospitalization code	45.9% (44.2%–47.6%)	4.7% (4.4%–4.9%)
	AECOPD identified using validated algorithm and hospitalization code	37.2% (36.3%–38.1%)	5.7% (5.4%–6.0%)

**Abbreviations:** CI, confidence interval; COPD, chronic obstructive pulmonary disease; AECOPD, acute exacerbations of COPD; LRTI, lower respiratory tract infection; HES, Hospital Episodes Statistics; FCE, finished consultant episodes; CPRD, Clinical Practice Research Datalink; PPV, positive predictive value.
